# A Mechanistic Theory of Development-Aging Continuity in Humans and Other Mammals

**DOI:** 10.3390/cells11050917

**Published:** 2022-03-07

**Authors:** Richard F. Walker

**Affiliations:** ProSoma, LLC, Clearwater, FL 33756, USA; drrwalker@gmail.com; Tel.: +1-727-422-6345

**Keywords:** developmental regulatory behavior, morphogenesis, morphostasis, morpholysis, initial conditions, determinism, chaos, aging mechanism, epigenetics, gene expression

## Abstract

There is consensus among biogerontologists that aging occurs either as the result of a purposeful genome-based, evolved program or due to spontaneous, randomly occurring, maladaptive events. Neither concept has yet identified a specific mechanism to explain aging’s emergence and acceleration during mid-life and beyond. Presented herein is a novel, unifying mechanism with empirical evidence that describes how aging becomes continuous with development. It assumes that aging emerges from deterioration of a regulatory process that directs morphogenesis and morphostasis. The regulatory system consists of a genome-wide “backbone” within which its specific genes are differentially expressed by the local epigenetic landscapes of cells and tissues within which they reside, thereby explaining its holistic nature. Morphostasis evolved in humans to ensure the nurturing of dependent offspring during the first decade of young adulthood when peak parental vitality prevails in the absence of aging. The strict redundancy of each morphostasis regulatory cycle requires sensitive dependence upon initial conditions to avoid initiating deterministic chaos behavior. However, when natural selection declines as midlife approaches, persistent, progressive, and specific DNA damage and misrepair changes the initial conditions of the regulatory process, thereby compromising morphostasis regulatory redundancy, instigating chaos, initiating senescence, and accelerating aging thereafter.

## 1. Definitions

The terms “*morphostasis*” and “*morpholysis*” are used for nomenclatural consistency with “*morphogenesis*” being the process by which systems change and evolve and “*morphosis*” being the sequence or manner of somatic development or change. Morphostasis refers to the transient post-morphogenetic period of young adulthood when the soma displays perfect adaptation and structural stability, absent morphosis, and frank aging. In humans, the period of morphostasis occurs during the decade immediately following completion of the last developmental stage. In contrast, “*maintenance*” refers to the energy expending process that avoids or repairs organismal damage occurring throughout life, but especially during and in opposition to the on-going effects of aging or “*morpholysis*”, the process of organismal deconstruction. Although often used synonymously with aging, and to describe certain cellular aspects of embryogenesis, “*senescence*” is defined as the point of transition between morphostasis and morpholysis when aging begins and exponentially progresses.

## 2. Introduction

Aging is generally thought to occur by programmed (adaptive) or non-programmed (non-adaptive/stochastic) processes [1,2]. Adaptive theories presume that aging evolved to benefit species, not individuals [3,4]. They provide reasons why, but not the mechanism by which, aging benefits evolution; however, its progression has often been said to appear programmatic.

Non-adaptive theories view aging as resulting from molecular damages that accumulates because chemical and physical constraints prevent their elimination by evolutionary processes. Stochastic damage can be quantified, and somewhat resisted, but cannot explain the programmatic patterns, sequences and timing of events associated with aging. Non-adaptive aging processes require “indirect” explanations, independent of a well-defined program [2]. 

The opinion that “impressive diversity” characterizes aging [2] leads to the erroneous assumption that its cause resides within the senescent phenotype. It does not! 

Each of the aging theories lack essential elements that are possessed by the other. Adaptive theories lack mechanism, while non-adaptive theories lack programmatic construct. The current theory provides both. Events that promote senescence occur “*within the scope of the developmental program*”, causing the progression of aging to appear programmatic.

The assumptions of the theory are as follows:

(a) A holistic regulatory program guides morphogenesis and morphostasis in the absence of aging;
(b) Purifying (stabilizing) natural selection sustains, but also threatens, regulatory program redundancy during morphostasis;
(c) Regulatory behavior during morphostasis changes with passage of time from causal determinism to determinative chaos;
(d) Progressively chaotic regulatory behavior erodes morphostasis redundancy initiating senescence and accelerating the rate of aging.

## 3. Theories Linking Development to Aging

Central to the theory is the developmental program (DP) which is universally accepted as “a cornerstone of modern biology that … drives all biological processes … from conception to reproductive maturation” [1]. This opinion leaves the question of what regulates the dynamic processes of living during young adulthood and beyond unanswered.

The notion that development is mechanistically linked to senescence has often been rejected in favor of separate aging programs [5,6,7].

A possible reason for this opinion is the mistaken impression that the DP has no purpose when somatic construction is complete. However, in humans, for example, morphogenesis ends with the emergence of an adult phenotype at about 20 years of age. Natural selection (NS) is still operable for about another decade, sustaining regulatory influence of the DP over the young adult soma to ensure maintenance of appropriate cellular ultrastructure and function as well as to complete its evolutionary obligation of successful reproduction. Other molecules that participate in development, e.g., morphogens/morphostats, also act in adulthood to maintain normal microarchitecture and to repair damaged tissues [8], while expression of developmental genes in adults suggest that the DP is operational throughout life [9].

Based upon this premise, several authors have offered theories linking aging directly to the DP [10,11,12,13,14,15,16,17]. 

While varying in specific details, all aforementioned theories assume that expression of the DP in some form continues beyond morphogenesis to cause or participate in aging. However, they are flawed because they assume that its continued expression into adulthood is maladaptive. If so, then its persistence into adulthood should have been selected against because the DP presumably ends in young organisms when NS is still operable. The lack of such post-developmental negative selection suggests that continuation of the DP during adulthood *has evolutionary benefit, at least initially*.

## 4. Is the DP an Aging Program?

Singer [18] proposed that aging is a “plastic” DP, encoded in the zygote, genome, and epigenome, whose expression begins at conception rather than after attainment of reproductive maturity. Presumably, variation in aging characteristics among individuals of the same species results from environmentally induced epigenetic changes, thus accounting for programmatic “plasticity”. He further proposed that interspecific differences in aging result from the expression of unique, evolved species-specific developmental programs, each with their own characteristic pattern [18,19]. Singer’s theory avoids the difficulties of explaining the evolution of maladaptive aging programs by making aging part of the universally accepted DP. Thus, he explains that aging emerges from the DP due to diminished strength of NS resulting from declining fertility, making it an “*integral part of the fabric of life*”. However, he does not explain why nor how the DP continues beyond its last developmental stage. This is a significant omission because essential to understanding the origin of aging is knowing how and/or in what form the DP continues after somatic construction is completed.

Instead, Singer proposed that programmatic aging is adaptive because it provides a means to “*regulate species population densities within the constraints imposed by the ecosystem organization*.” [19]. This concept is similar to Mitteldorf’s demographic theory [20] and may well provide an evolutionary explanation for the benefit of aging in regulating population density. However, it does not identify a discrete aging mechanism or provide convincing evidence that demographic stability was the primary factor for selection of developmental “plasticity”. Singer [21] further claimed that biological processes are not uniquely different in young and old organisms, thereby providing “*considerable evidence linking aging to developmental programs*”.

In agreement with Singer, the current theory accepts that the mechanism linking development to aging originates at conception but rejects the concept that biological processes specific to organismal aging do not exist. Nonetheless, if species-specific developmental programs have different trajectories, then they may also have related, but dissimilar, mechanistic properties. If true, the exact descriptions of shared aging mechanisms among species would be difficult to achieve. Thus, for interpretive accuracy, the main focus of the current mechanistic theory is upon mammalian aging, especially the human condition. 

## 5. Development

Development is defined as “the series of progressive, nonrepetitive changes that occur during the life history of an organism. The essence of this definition contrasts development with metabolism, the essentially repetitive chemical changes necessary for day-to-day functioning of the body” [22]. 

These definitions raise questions about how the mechanism for senescence begins and organismal aging proceeds from development, including:

(a) Does metabolism play any role in aging, i.e., is progressive metabolic insufficiency a cause or consequence of aging?
(b) What, in general, are the events regulating the dynamic process of development at each stage of the DP?
(c) Are stages of the DP truly nonrepetitive, or do one or more continue to be expressed upon completion of morphogenesis? If so, for what purpose and for how long?

## 6. Metabolic Thermodynamics

Yates described conversion of energy in biological systems at various life stages to provide a homeodynamic overview of how development and aging are related [23]. 

Upon initiation of development, self-organizing systems produce new forms and functions from internal energy. The complexity of morphogenesis increases with each developmental stage, as internally negentropic transactions reflect the increasing order. However, upon completion of human morphogenesis at approximately 20 years of age, adulthood begins. Thereafter, many of the previously available degrees of freedom that were needed to complete development are unavailable. This restriction constrains further energy transformations from a constructive to a maintenance scenario, thus providing a clue to understanding how DP regulatory oversight changes after completion of somatic development.

Before “*senescence begins*” in humans at approximately 30 years of age, Yates represented flows and transformations of energy as “*open thermodynamic systems* [that] *necessarily organize energy processing as cyclic physical action modes.*” These are envisioned each day metaphorically as a helix so that, as senescence emerges, the amount of Gibbs’ free energy that can be extracted each day “*surreptitiously decreases*” [23]. Thus, internal entropy increases as coherence and complexity are lost due to the breakdown of *regulatory oversight and control* [24]. Subsequently, the area of the metaphorical helix decreases, transforming into a progressively truncating ellipse that eventually closes, thereby effectively setting the lifetime energy budget [23]. As that limit is approached, energy throughput slowly erodes order causing the organism to become fragile, vulnerable to intrinsic disease and eventually to succumb [25].

Thus, Yates [23] concluded that “*the central and most general physical cause of senescence (i.e., aging) is diminishing energy throughput*” resulting from the decay of regulatory processes. While being the energetic basis of mortality, progressive metabolic insufficiency is a consequence and a contributing factor for aging, whereas gradual loss of regulatory oversight and control is its proximate cause.

Consistent with Yates’ thermodynamic application of the second law to aging, Hayflick [26] explained that age changes occur spontaneously due to irreparable losses in molecular fidelity, not governed by genetic influence. No reference was made to regulatory breakdown or any other possible process as a contributing factor(s) for molecular instability that initiates senescence. However, he suggested that strong element of aging uniformity exists because errors occur first in the same families of most vulnerable molecules in similar cells, organs, or objects. Thus, a “*weakest link first to fail mechanism*” accounts for similarity in the aging phenotype as it progresses within species members [1,26]. However, the “*weakest link first to fail*” process implies sequential expression of the aging phenotype, which is inconsistent with the generally accepted view that aging simultaneously affects all parts of the body [11].

Since energy processing is not the same throughout the body, spontaneous decay of a specific metabolic pathway(s) cannot be a primary factor subserving organismal senescence. Instead, metabolic collapse occurs throughout the body, secondarily to some initiating event. The simple basis for this speculation is that caloric restriction (CR), similar to aging, *diminishes energy throughput* but extends rather than shortens lifespan. This observation suggests that by reducing caloric intake to a level above starvation, food restriction and resultant damping of molecular thermodynamics slows down the expression of damage associated with transformation of the entire body proportional to the age at which it is begun [27]. Nonetheless, death is inevitable, albeit occurring later than it would if food were available ad libitum. Thus, organismal senescence requires maladaptive, energy-dependent events whose rates of occurrence can be thermodynamically modulated. For example, certain agents that attenuate constitutive levels of mTOR signaling, thereby presumably reducing metabolic activity, concomitantly lower DNA damage from endogenous reactive oxygen species which can extend life [28]. More relevant to the theory is that CR-diminished DNA double-stranded breaks (DSBs) while extending life span [29].

Without describing the series of events responsible for age-related breakdown of regulation, Yates provided insight into developmental stage dynamics and how they participate in the emergence of morphostasis from morphogenesis, the initiation of senescence, and the acceleration of morpholysis from morphostasis. Accordingly, attention is now turned to regulatory behavioral dynamics as they direct events subserving the continuity of development and aging.

## 7. Regulation of Organismal Development

The DP is recognized as an evolved biological program within which interacting processes create heterogeneous shapes, sizes, and structural features on a dynamic trajectory from embryo to adult [30].

Clues to understanding the relationship between development and aging exist in *Morphogenesis: An Essay on Development* [31]. At the time of his work, recognizing the growing amount of data on developmental phenomena, Bonner realized that there was no unifying theory with which to interpret them. This observation is not unlike the current situation in aging research, where the assumed complexity of the aging process has been an insurmountable barrier to identifying a specific mechanism that regulates its origins and progression. Kirkwood [32] suggested that data on aging have grown in a patchwork way, through the pursuit of specific single-aspect theories. He felt that, after many decades of such pursuit, single-aspect theories have limited explanatory power, such that interacting theories may be better analyzed using methods that have the power to determine how its apparent cumulative effects cause aging. 

Accordingly, he suggested the application of network theories to determine how individual mechanisms interact with each other [33,34]. 

Unlike contemporary biogerontolotists, Bonner [31] saw greater promise in examining aspects of the *total organism* to unify developmental theory. Morphogenetic stages are identified as products of structural transformation guided by DP regulatory mechanisms. Twenty-three stages/modules of embryogenesis are recognized [35,36], beyond which stages of development continue until constructive stages of the DP end. Somatic maturation occurs in humans at approximately 20 years of age [37]. 

While successfully describing progression of many aspects of total embryonic development, Bonner recognized that “*regulation*”, a most elusive quality, was required to unify the stages of structural transformation into a coherent integrated soma. This was an important issue since developmental regulation makes the coordinated transformation of the entire organism possible during all stages of morphogenesis, allowing it to *function as a whole throughout development*. It is important to stress that despite exponentially growing knowledge of individual constructive and maintenance metabolic pathways, none were known to display such holistic influence. Bonner and others before him were frustrated by their inability to provide a scientific explanation for the phenomenon. The mysterious nature of organismal developmental regulation inspired Hans Driesch, a biologist, early embryologist, and philosopher, to explain it using the neo-vitalist philosophy of entelechy [38]. Knowing that all cells in the body contain the same genetic code despite each having different structures and functions, Bonner was unable to speculate on how a synchronic informational genotype could functionally interact with a diachronic dynamic phenotype. So, to avoid potentially “supernatural” aspects of vitalism, he simply concluded that organismal development results from the dynamic interaction of ever-changing constructive and limiting processes. 

Prior to the work of Waddington [39,40,41], the difficulty Bonner experienced in explaining “*regulation*” derived from the paucity of information on how gene expression is influenced by internal and external environmental “epigenetic” factors. Although epigenetics has been a rapidly growing field of study, it took almost two decades for evidence to emerge around DNA methylation and its developmental role to switch genes off and on [42]. A functional relationship between DNA methylation and gene expression emerged during the 1990′s [43] when it was recognized that patterns of gene expression differ greatly among different cell types, while all of an organism’s somatic cells contain the same genome. More recently, the process of activating or silencing genes was discovered through the study of certain chemical tags or epigenetic modifications that attach to different parts of DNA and its associated proteins. Thus, while not altering the underlying genetic code, the epigenetic process controls gene expression, thereby affecting the proteome, specific structures, and functions of different tissues.

Thus, when Bonner was creating a unified hypothesis for developmental biology, knowledge of epigenetics was just emerging. It was not until decades later that its involvement in gene expression was better understood. This lack of information contributed significantly to the difficulty in understanding global somatic regulation, i.e., how information within the genotype which is the same in all cells can be translated into a changing phenotype. Had epigenetics been better understood during his time, Bonner may have found a more satisfactory explanation for the “*mysterious*” and special biological force of organismal regulation [31,38]. 

## 8. Global Influence

Questioning the conventional wisdom that the aging process emerges from an amalgamation of random, detrimental effects, Ohsawa et al. [44] suggested that particular cellular pathways affect its existence and expression. The challenge was to understand the mechanistic basis of how such pathways and metabolic states regulate aging.

The complexity of organismal aging and development derives from the multitude of molecular constituents and robust processes that interact across many spatio-temporal levels. That robustness suggests *the existence of relative simplicity in holistic oversight of developmental and aging dynamics, perhaps involving global constraints that ensure similar outcomes despite fluctuation in the underlying mechanisms* [45,46]. Thus, it is assumed that multiple mechanisms of the soma are subject to regulatory oversight by a process that consolidates them into a single program.

An important premise of the current theory is that the global key for regulating development and aging resides within the genome and hence, is present in all parts of the body. This life-long influence over the total organism in one form or another is a key factor linking developmental “*regulation*” to the mechanism of aging. Upon completion of sequential, non-repeating developmental stages, the expression of the regulatory process is modified during morphostasis to appropriately direct dynamics of the stable, young adult organism. While adaptive was selected, this modal alteration of expression beyond morphogenesis presents a condition within which regulatory behavior favoring the potential for emerging senescence exists. 

The global regulatory influence of the DP is particularly relevant to formulate a unified aging theory because senescence “*should always be a generalized deterioration, and never due largely to changes in a single (physiological) system*.” [11]. Williams and others [11,47,48] stressed that senescence simultaneously affects all organs and systems “In mammals and especially man”. Medawar [49] agreed but stipulated that among the multitude of changes associated with aging, gerontologists should distinguish cause from effect, so as to identify one or perhaps a few ultimate causes. Williams disagreed, claiming that “*such small number of primary factors is a logical impossibility*”. This assumption is consistent with contemporary views that aging is multifactorial. Williams [11] was correct in assuming that of all the maladaptive, age-related, changes in gene expression occurring throughout the body during aging, no specific one or few is responsible for causing it. However, he was incorrect in assuming that *the multitude of changes associated with aging* are causal, not consequential. He also failed to consider the possible role of developmental regulation in aging. In contrast to the concept that aging is multifactorial, failing regulatory oversight of post-maturational morphostasis cycles is a singular source of senescence, as described in the current theory. 

The relationship between aging and the DP has received little attention despite reports of progressive, age-related, seemingly “programmatic” changes that occur in humans beyond age thirty and at appropriate ages in other species that nurture their offspring [11,50]. This characteristic of post-developmental regulatory dynamics could be viewed as prima facie evidence for the existence of an aging program. Depending upon interpretation of the theory’s supporting data, it may be; however, then again, it may not. 

“*Regulation*” has been overlooked by contemporary biogerontologists as playing a central role in the aging process. The reason for this oversight is perhaps the difficulty in grasping the concept that one process can not only sequentially regulate construction, but also transient stability and deconstruction of the complete soma. Examination of developmental regulatory dynamics at various stages of life provides an understanding of this seemingly paradoxical relationship.

## 9. Regulation of Non-Repeating Developmental Stages

An important first step in understanding the continuous relationship between development and aging is to consider that “*regulation*”, which begins within the DP at conception is a process that is expressed during the procession of life unto death. That process not only evolved to direct construction of the specific products of each developmental stage, but also to preserve the post-morphogenetic stage of morphostasis for as long as possible. It is necessary that the character and expression of regulatory oversight be altered in order to appropriately oversee successful progression and completion of those two different DP processes.

Upon conception, development begins during an initial embryonic stage(s) that forms tissue patterns and novel structures as products of autonomous, self-organizing cellular mechanisms [51]. Thereafter, in compliance with regulatory oversight, complexity of morphogenesis increases.

A pattern-to-pattern characteristic of developmental stage progression could allude to the order of somatic construction, i.e., the “assembly of parts, or the particular arrangements of cell states in three-dimensional space” [52]. It could also describe the dynamics of regulatory events that direct appropriate synthesis and assembly of parts during each stage. The latter case subserves the regulatory process of development-aging continuity.

Regulatory behavior which governs the progression of each developmental stage is an important *process*, the outcome of which relates to specific *products* that are appropriate for the local assembly of structural “parts”. The products of each developmental stage are emergent molecules that interconnect by providing a substrate for the synthesis of novel structures for the next developmental module. They not only act as a substrate for continued development, but also serve an epigenetic function which, in conjunction with other epigenetic modifiers, determines regulatory gene expression while constraining behaviors and directions of events associated with a novel outcome for each developmental module. Epigenetic influences derived from previous stage products, as well as within-stage gene expression, carry great weight in affecting product formation of any given stage [53]. This important factor affecting regulatory “behavior” is central to understanding the emergence of senescence from the redundant stage of morphostasis.

Each developmental stage follows a general pattern due to oversight by the same, albeit epigenetically modified, sequence of regulatory factors. Thus, one stage follows the other, not only in numerical sequence, but by being connected through the sharing of the substrate and essential epigenetic information required for proper gene expression and progression of the complete morphogenetic series. This process causes each stage of somatic construction to consist of different structural parts that are linked pursuant to guidance by the same “basic” regulatory sequence.

In addition to the synthesis of new parts, a core concept of the present theory is that the basic “*chain of march*” or specific sequence of genetic components within the regulatory process that directs phenotypic alteration never changes. Unlike the patterns of *construction* that change phenotype from stage to stage, the basic underlying pattern of *regulation* stays the same, except that epigenetically influenced, qualitative changes in expression of common regulatory *sequence* components, produce specific products appropriate for physical construction of each body part. Thus, while regulatory sequences or execution *patterns* directing homeodynamics during stages of development are similarly constructed, their components vary qualitatively at each developmental module, causing the outcomes to be different. 

Thus, somatic development cannot be isomorphic with a defined programmatic structure, but instead unfolds more “*like the development of an ecosystem in which* [after process initiation] *events follow a predictable* [but not exact] *sequence in the absence of any program.*” [23,54]. Given the lack of specific and necessary regulatory information required for completion, additional guidance for structural transformation comes from actions within and interactions between developmental modules. Developmental restrictions [55], as well as bias, do not limit the phenotypes available for selection of a specific design or product at each stage of somatic construction accounting, in part, for different developmental trajectories among even closely related individuals. Since the DP progression follows a pattern to pattern format, each stage occurs only once. Furthermore, because it is non-repeating, it does not progress to *chaotic behavior that is at the heart of the aging mechanism.* Instead, each DP stage is finite and initiated by qualitatively different factors, consistent with *determinative behavior*, which prevents aging during development, as discussed later in greater detail.

Thus, based upon differential dynamics of the developmental regulatory mechanism, human beings look like others of their species, but none are identical. Even monozygous twins are not exactly the same due to differences in modifying epigenetic components of the same regulatory operational pathways, albeit small, which cause different outcomes nonetheless [56]. Individual organisms undergo multiple changes in response to independent gene actions, as well as *their internal states and external environments* that alter and shape their unique developmental trajectories [57,58,59]. Because these cooperative and combined effects are not specifically programmed, each developmental unit is “execution-driven” as much as it is “program driven” [60]. This processing explains how the different stages of development can emerge from a single but ubiquitous, genetic, regulatory “backbone” or sequence. Functional phenotypes resulting from such combined genetic *and* epigenetic/environmental influences promote evolvability and thus favor evolution [61,62,63]. 

The relevance of the developmental regulatory process to the theory relates to the fact that the product of one developmental stage epigenetically affects expression of the next initiating gene(s) in the construction sequence for each particular cell type. Both product and expressed gene(s) create the *initial conditions* from which the next stage proceeds. Since the non-repeating stages of morphogenesis end upon somatic maturation, the regulatory process must be appropriately modified to accommodate the absence of emerging new structures during morphostasis. 

## 10. Redundant Expression of the Last Morphogenetic Stage

It is during the last morphogenetic stage, upon initial emergence of the adult phenotype, that non-repeating stages of morphogenesis end along with non-repeating qualitative changes in its tissue specific regulatory components. Some investigators proposed that when the DP ends, one or more unprogrammed stages could continue to be expressed thereafter, but without purpose. Presumably, such continuation of various processes associated with the DP into adulthood, cause somatic destabilization and initiate senescence. Magalhães [10] proposed that during the expression of the DP, a continuation of patterns that are adaptive during development become maladaptive in adulthood, eroding somatic integrity and causing aging. He explained that overexpression of developmental patterns occurs because a “*short-sighted watchmaker*”, i.e., evolution, responsible for creating the DP inadvertently overlooked the need for its eventual termination [10]. Consistent with theories of others, over-expression of the developmentally related events presumably alters outcomes of post-morphogenetic, regulatory signaling, thereby eroding somatic form and function for any number of possible reasons previously cited [11,12,13,14,15,16,17]. There are several basic problems with such hypotheses. 

First of all, just as “*God does not play dice with the universe*” [64], so “*Evolution does not overlook essential details of its creations*” [author’s opinion]. Continuation of maladaptive developmental “construction” patterns into adulthood is structurally improbable because the progression of morphogenetic stages follows a pattern-to-pattern process, whereby the product of one stage initiates the next, as does its product with the next, etc., until development is complete. Thereafter, no novel emergent products remain to initiate additional developmental modules and outcomes. Since no adaptive purpose would be served if they were part of a developmental program that presumably completed the evolved task of somatic construction, they would not occur. If they inadvertently did, they would be selected against because NS is still influential when morphogenesis ends at about the human age of 20 years. Moreover, somatic construction and developmental transformation of the soma cannot continue indefinitely because physical limitations, morphogenetic construction rules, phyletic developmental constraints, laws of diffusion, hydraulics, and physical support prevent it [55]. Finally, once development is complete, signaling pathways for mechanisms regulating novel pattern formation are typically inactivated, thereby preventing further developmental change in structures related to morphogenesis [65]. 

Thus, one might expect that the DP ends upon completion of its last stage. However, if “*Nothing in biology makes sense except in the light of evolution*” [66], as Dobzhansky confidently stated, then termination of the DP and its “regulatory process” would also be expected to end. If so, then the integrated workings of evolution would seemingly cease to be involved in the dynamics of living beyond morphogenesis into young adulthood. As a result, the post-morphogenetic, expression of morphostasis, senescence, and accelerating aging would lack global influence, and thus “*not make sense*”. Importantly, termination of the DP would prevent initiation and control of somatic morphostasis which was selected to ensure completion of the evolutionary obligation of reproduction in species whose offspring require parental nurturing. 

Understanding the need for regulatory oversight while accepting that construction of *novel* products from randomly continuing, non-repeating developmental stages beyond morphogenesis would be improbable; thus, the DP must continue in an alternative form. Upon completion of development, dynamic degrees of freedom for further construction of the soma are “frozen out”, causing “*the series of progressive, nonrepetitive changes occurring during morphogenesis*” [22] to end. To do so requires limiting and redundantly expressing DP regulatory oversight to those functions, as performed during execution of the last developmental module. These constraints on regulatory expression prevent production of new structures and temporarily maintain the young adult phenotype. It is during this pre-senescence period that Yates [23] metaphorically described daily energy processing as helical, i.e., cycles within which the risk for expanding internal entropy increases. 

For the last stage of the DP to become redundantly expressed, its regulatory process must be modified to comply with the rules that evolved for proper guidance of each morphogenetic stage. The regulatory process of developmental stage progression is initiated by the emergent structure of the preceding developmental stage that not only provides a structural basis for assembly of the next product, but also epigenetically affects expression of the initiating regulatory gene(s) for that stage. It is the combination of these epigenetic and genetic influences, along with other environmental factors that establish the “initial conditions”, that determine how that stage will proceed. During maintenance of morphostasis, deterministic behavior is strictly dependent upon precise redundancy, which will degrade if conditions for its maintenance are disturbed. 

To accomplish and sustain redundancy while employing the established regulatory process, the product of each morphostasis cycle must be the same as the one originally produced by the last morphogenetic module. Thus, the final product of the morphogenetic stage then becomes the initiating product of each subsequent, redundant morphostasis cycle. Thus, during the second iteration of the regulatory process governing the last developmental stage, its product becomes the epigenetic influence that directs gene expression to produce the same products of the preceding cycle. Thus, redundancy occurs to maintain fidelity of the young adult soma as each cycle is precisely re-expressed. Similarly, expression of the same initiating gene(s) would be epigenetically affected as during the first iteration of the last DP stage. As a result, the initial conditions would be replicated for each successive cycle, thereby allowing morphostasis to continue in the absence of aging for a period of time lasting in humans for approximately the first decade of young adulthood.

As generally recognized, the “genetic program the genome is optimized for reproduction… including child rearing” and the “care of dependent progeny is as important to reproduction as gamete production” [10,11]. Thus, besides birthing, mammalian reproductive success requires parental devotion of relatively long time periods to nurturing, protecting, and educating offspring before they become independent. Since evolutionary obligation in mammals demands that young adults remain viable, fit, and capable of providing their progeny with essential requirements for survival until independence, it is logical that a mechanism to sustain such prolonged vitality evolved as part of the DP. 

Because redundant expression of the last developmental regulatory process was selected to initiate and temporarily sustain the adaptive stage of morphostasis during the first decade of human adulthood, the opinion that the DP no longer affects the soma beyond morphogenesis is incorrect. Its regulatory influence continues for the remainder of life. However, beyond NS, redundancy progressively fails, altering behavior of the regulatory mechanism from causal determinism to deterministic chaos. It is important to note that the behavior of the regulatory process, whether in non-repeating or redundant stages, is malleable, and though its behavior may change throughout life, it continues oversight of structural modification, albeit to the soma’s detriment beyond morphostasis, i.e., during morpholysis. 

This behavioral change causes aging that emerges within the failing regulatory apparatus to appear “programmatic”. Furthermore, increasing loss of regulatory redundancy increases chaos over time, thereby also accelerating the rate of aging [67]. Thus, the regulatory process, albeit increasingly damaged over time, remains linked with the last developmental stage throughout life. 

## 11. Molecular Components

The molecular construct of the regulatory mechanism contains a common and specific “*genetic backbone*” which exists as part of the genome and, hence, is located within all cells of the body. Thus, its global distribution accounts for the ability of the regulatory process to influence the complete soma simultaneously and throughout life. Specific expression of the regulatory genes is affected by the epigenetic landscape(s) existing in the multitude of different somatic cells and tissue types of the organism, as well as by the product of the preceding stage, making them interdependent components of phenotype regulation. Gene expression is also influenced by chromatin remodeling, environmental and other local epigenetic factors, as well as DNA damage repair. Thus, while each stage of a developmental sequence may not be specifically orchestrated, there are genetic, initiating events in concert with the product(s) of the preceding stage that set the epigenetic landscape for the current cycle. In this way, genetic and epigenetic actions “influence” the general direction of somatic transformations, but not their specific trajectories. Because of the differential influences of initiating and emergent epigenetic factors, genes are expressed differently across stages of life [53].

While developmental regulatory events occur simultaneously to coordinate actions throughout the total organism, the signaling processes that direct integrated functions remain unknown. However, it is reasonable to speculate on the general composition of the global regulatory mechanism that oversees phenotypic change across the span of a lifetime. 

In his discussion of regulatory control of morphogenesis, Yates implied that the pattern-to-pattern sequencing of developmental stages is guided by a common regulatory mechanism initiated by “*…**genes** [which] act as dynamical constraints shaping **product formation** at each stage [that]…**act as new constraints** on the next round of dynamics. **Epigenetic** influences carry great weight…*” [68]. 

If the regulatory system affects phenotypic changes across the lifespan, and since the genome is the same throughout the body, it must be the unique epigenetic environments of the various cells and tissues that affect differential expression of genes, both temporally and appropriately for their geographic (spatial) locations. Thus, epigenetics makes the essential contribution that explains how a constant or fixed genotype could direct the various changes in phenotypes that occur during the transition from development to advanced age, i.e., how a synchronic informational genotype could functionally interact with a diachronic dynamic phenotype.

The theory assumes that initiation of each regulatory cycle begins with the epigenetic product of the preceding stage in conjunction with non-coding DNA (ncDNA), to which a major portion of transcriptional activity in mammalian cells is attributed. It is proposed that ncDNA participates in the process of developmental stage initiation because it contains sequences that act as regulatory elements to determine when and where genes are activated or not, and provides sites for transcription factors to bind and either activate or repress transcription. Additionally, regulatory elements of ncDNA include promoters, enhancers, silencers, and insulators, and also provide instructions for the formation of certain kinds of RNA.

Although ncDNA does not code for proteins, its transcription occurs throughout eukaryotic genomes, generating a wide array of ncRNAs [69] that account for a major portion of the transcriptional activity observed in cells. Some of these affect genes and interact with protein complexes to modify chromatin structure [70], demonstrating their importance as regulatory molecules. One large class of ncRNAs includes those transcribed over the promoter regions of nearby protein coding genes. As a result of these important roles, ncRNA molecules have been considered by some to be genes [71] that play an important role in an epigenetic network, thereby highlighting their prominent regulatory role [72]. 

The assumption that ncDNA is part of the initiating sequence for each regulatory cycles is also based upon the experimental evidence from Hayano et al. [50] who reported on a murine system within which endonuclease-induced DNA damage, i.e., non-mutagenic double-stranded breaks (DSBs), could be precisely controlled at frequencies only a few-fold above spontaneously occurring, normal background levels. The DSBs that were created primarily in non-coding regions altered the epigenome while initiating and accelerating organismal aging in young adult laboratory mice [50]. 

Both genetic and epigenetic factors set the initial conditions for the newly beginning regulatory sequence. This is an important role, since the regulatory behavior beyond morphogenesis during morphostasis and morpholysis observes laws of determinative chaos (DC) and, thus, displays sensitive dependence upon initial conditions (SDIC). Subsequently, ncRNAs that are transcribed from the ncDNA to further affect gene expression modify the chromatin structure by interacting with protein complexes that further establish and maintain specific epigenomic landscapes [69,72,73,74,75]. Additionally, some sections of ncDNA transcribe ncRNA over promoter regions where they affect coding gene expression and play important roles in post-transcriptional regulation [69,76,77]. Thus, they initiate expression of protein coding genes as a secondary function of the initial events. Coding genes then direct production of essential proteins, explicitly specifying their primary structures. Thereafter, epigenetic factors existing as parts of the spatial and temporal environments create dynamical constraints on a subsequent higher-order protein structure, folding, targeting, scaffold attachment regions, origins of DNA replication, centromeres, and telomeres. Since coding genes are played upon to produce appropriate products for construction, they are not likely to be part of the initial conditions in the regulatory sequence for each developmental stage. Coding genes do have regulatory sequences but they are used to control protein production, not to maintain a stable young adult soma or sustain youth during morphostasis. Thus, it would seem that if coding genes are responsible for creating structure, i.e., protein, no individual one or combination could be a primary regulatory gene responsible for the establishing patterns that occur during the aging process. 

The uniqueness of “regulation” that guides developmental stages and morphostasis throughout the entire organism is proposed as having the potential to similarly affect organismal aging as the life cycle transcends the limits of the DP morphostasis component (initial stage of adulthood) in order to experience senescence and accelerate aging (midlife and advanced age) and its challenges.

## 12. Determinism and Chaos

The holistic organismal regulatory mechanism described herein can sustain somatic integrity, health, and vitality, or can cause disorganization, dysfunction, and failure depending upon the behavioral conditions under which it is operating. Causal determinism (or determinism) avoids aging during morphogenesis and precisely redundant morphostasis, whereas loss of redundancy causes determinative chaos (or chaos) to emerge, allowing senescence to begin and morpholysis to proceed exponentially.

**Figure 1:**
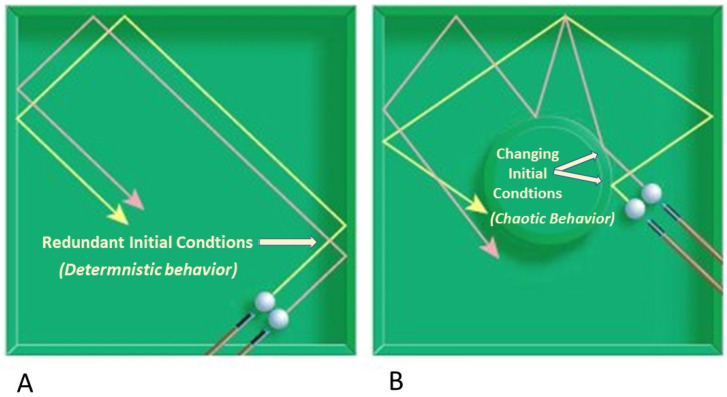
Visual metaphor of developmental regulatory behavior. (**A**) Causal determinism: initial conditions are redundant when balls strike a flat surface, resulting in the predictable outcomes. Rebound angles will repeat the initial conditions. Similarly predictable regulatory behavior occurs during non-repeating and strictly redundant stages of morphogenesis and morphostasis, respectively; (**B**) deterministic chaos: initial conditions become slightly divergent but somewhat predictable when balls first strike a convex surface. However, subsequent rebound angles become increasingly unpredictable as chaotic behavior increases. Similar changes in regulatory behavior occur during late morphostasis and morpholysis as senescence emerges and aging accelerates, respectively. *Figure 1 is licensed under Creative Commons Attribution 2.5 Generic license and made available free of charge to adapt the work as it relates to the current Theory. This statement is made to confirm that the licensor does not endorse the current author or his use of the Figure*.

Determinism states that every event (*effect*) is influenced by its antecedent events (*cause*) and conditions consistent with the laws of nature [78]. In other words, the precise combination of regulatory events at a certain time will result in a predictable outcome. To the extent that a system can be perfectly isolated when identical starting conditions are repeatedly imposed, its subsequent behavior should be *determined by those initial conditions and exhibit unique evolution* (Figure 1A). 

Thus, progressive elements that are determined by initial as well as boundary conditions follow a common sequence and, importantly, are finite or non-repeating [22]. In biology, determinism is applicable during morphogenesis and redundant morphostasis, but for different reasons. In the first case, non-repetition of developmental stages prevents chaos from developing, whereas chaos is avoided during morphostasis, at least temporarily, by the precise redundancy of the last developmental stage.

In contrast to determinism, deterministic chaos [79] presents the paradox of linking two seemingly incompatible notions of predictability or determinism with unpredictability. Chaos begins with gradual erosion of regulatory redundancy during late stage morphostasis, thereby initiating senescence.

While chaos describes a lack of predictability, it does not mean randomness, which can produce an ensemble of different outputs from the same set of parameters and initial conditions. In contrast, chaotic systems are deterministic, dynamic, and nonlinear [80]. Because they display exquisitely sensitive dependence upon initial conditions (SDIC), have patterns, and lack random components, they are quite ordered and even predictable on *short time scales*. Lyapunov time is the characteristic time scale upon which a dynamical system becomes chaotic, thus mirroring the limits of predictability [81]. In practice, a meaningful prediction cannot be made over an interval of more than two or three times the Lyapunov time. However, small changes at the start of a chaotic process that are repeatedly expressed will exponentially degrade predictability over extended intervals of relative time to produce complex, long-term behavior that causes unpredictability to emerge [82]. Aging displays such chaotic behavior by creating trajectories that are unique to each individual. Predictable changes occur early on; however, with time, dissociation of trajectories, even among closely related individuals, diverge and become unpredictable. The empirical observation of very old (>90 years) humans suggests that an interval that is at least twice the Lyapunov time makes the prediction of future events seemingly random and unpredictable. Uncertainty of forecast increases exponentially during aging where the effect of chaos is consistent with predictions of the Gompertz function [34]. Thus, the alteration of initial conditions, however small, is persistently magnified by dynamics of the system, causing exponential amplification of errors. Two nearby initial conditions in generic position will give rise to trajectories that separate with time, amplifying small errors (Figure 1B). As time passes, the system “appears” to behave randomly, such that meaningful predictions can no longer be made [83]. Such behavior that looks out of control but which functions according to unseen rules or organization has been defined as “*stochastic behaviour occurring in a deterministic system*” and is sometimes called ‘*constrained randomness*’ [84]. Because SDIC is crucial in chaos theory, it has been incorporated in similar concepts by other authors who intend to explain the dynamics of aging [67]. The apparent randomness, particularly related to ageing, can be interpreted in the light of chaos theory [85,86,87]. This is an important issue relevant to the divergence of characteristics that occur even among closely related individuals, such as monozygotic twins as they grow older and older, representing the root of unpredictable aging trajectories.

In light of the relevance of the current theory, it is not chaos that initiates changes in initial conditions. Instead, it becomes a dominant behavior of the regulatory system once morphostasis redundancy is lost due to damage-based structural change that alters initiating genes and thereby outcomes of each subsequent cycle. As this occurs, the influence of the remaining redundant regulatory cycles of the last developmental stage continue to be expressed. However, their effect to sustain a youthful phenotype becomes progressively degraded. As damage quantitatively reduces the number of genes with appropriate structure to provide correct initial conditions, accompanying epigenetic changes accrue causing chaos to exponentially degrade the phenotype.

## 13. Characteristics of Determinism and Chaos

Persistent determinative behavior remains predictable (Figure 1A). This property is not inconsistent with systems that exhibit chaotic behavior so long as there is SDIC. Although initially predictable, when conditions change over sufficient time, the behavior of chaotic systems eventually appear to behave randomly relative to the starting conditions (Figure 1B).

The shifting influence of causal determinism to deterministic chaos during the changing expression of developmental regulatory dynamics underlies the emergence of senescence from morphostasis and eventually to the exponential acceleration of aging (Table 1).

**Figure 2:**
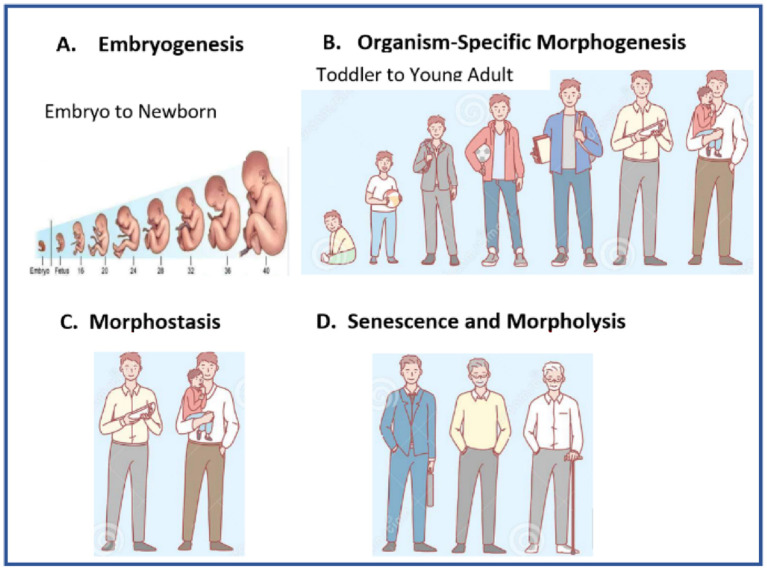
Stages of life and associated organismal regulatory dynamics. (**A**) **Regulatory Stage 1:** Qualitative and quantitative increases in holistic regulatory mechanism components. **Number and type of regulatory processes increase during embryogenesis as anatomical structures are generated and organized producing novel epigenetic landscapes in newly forming cell and tissue types.** (**B**) **Regulatory Stage 2:** Qualitative changes in holistic regulatory mechanism upon completion of embryogenesis. **After eight weeks, the embryo has created all organs and tissues of a newborn baby; however, many are primitive.** Thus, the maturational process of organs and tissues modifies the epigenetic landscape for appropriate gene expression and regulatory guidance of the soma unto organismal maturation which occurs at about 20 years of age. Regulatory behavior during morphogenesis is deterministic; thus, aging does not occur. (**C**) **Regulatory Stage 3:** Non-aging decades of offspring nurturing, during which genetic and epigenetic changes in the organismal regulatory mechanism do not occur or are minimal because guidance of the last morphogenetic module is redundantly expressed. Strict redundancy of regulatory mechanism dynamics sustains deterministic behavior and thereby avoids aging. Toward the end of the post-maturational decade as the strength of natural selection wanes, DSBs cause gene structural errors and subsequent epigenetic changes that initiate loss of regulatory redundancy and change its behavior to deterministic chaos. (**D**) **Regulatory Stage 4:** Maladaptive, qualitative changes in genetic and epigenetic components of the holistic regulatory mechanism progressively increase the loss of its redundant expression, thereby exacerbating chaotic behavior which accelerates aging.

A diagrammatic representation of dynamic changes in the organismal/holistic regulatory mechanism from embryo to old age (Figure 2). 

## 14. Maintenance vs. Morphostasis

Maintenance has been described as the avoidance or repair of *age-related* damage through energy expending processes, excluding those for somatic construction. These temporarily preserve tissue integrity and extend adult organismal lifespan [88] in opposition to the age-promoting, maladaptive effects of concomitantly decaying morphostasis regulation.

Based upon Monte Carlo modeling [89], Rozhok and DeGregori [90] proposed that a somatic maintenance program (SMP) evolved specifically to enhance survival. In addition to the SMP, other processes in aggregate, such as various tumor suppressor gene functions (including DNA damage-induced apoptosis), autophagy, purifying somatic selection, immune surveillance, and morphogen-like controller molecules called morphostats [8], also participate in promoting somatic maintenance, thereby extending lifespan.

**Figure 3:**
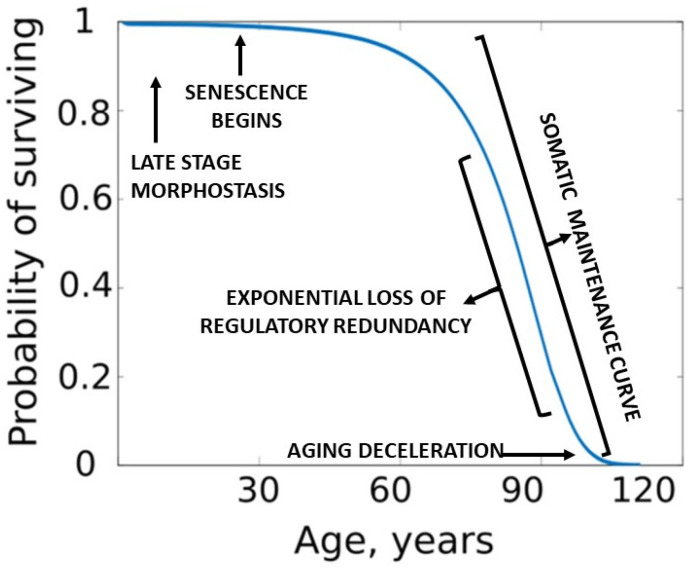
The SMC representing the combined effects of somatic maintenance and loss of morphostasis redundancy on modern human mortality in the USA. The probability of human survival just prior to 30 years of age is the essentially the same as during the preceding decade. Increasing loss of morphostasis redundancy accelerates the rate of aging which is opposed by somatic maintenance. Upon exhaustion of redundancy, the rate of aging decelerates due to the continuing influence of maintenance. *Figure previously published by* [90] *reproduced with modification pursuant to Creative Commons Attribution 4.0 International License (http://creativecommons.org/licenses/by/4.0/)*.

Assuming that somatic maintenance is active during the period of exponentially increasing death rates, and since human and captive animal mortality curves approximate the physiological aging curve, the authors felt that opposition to aging via the SMP could be graphically represented as an inverse plot of the Gompertz function [91] which begins in humans at approximately age 30 [92]. Rozhok and DeGregori [90] called the inverse of the Gomperz function a “*somatic maintenance curve*” (SMC), which presumably describes the interaction of age-promoting accumulation of structural damage due to redundancy loss and the SMP that opposes those effects. (Figure 3).

Resistance to the progression of aging by maintenance is acknowledged as a life extending process. However, it is quite different from the unique programmed process of morphostasis, during which the young adult soma is temporarily maintained *in the absence of aging.* The relationship of somatic maintenance and morphostasis is presented in Figure 3. It is worthy of note that life table aging rates suggest deceleration of mortality at very old ages [93]. One interpretation of these data could be that maintenance improves or is more effective in those living to extremely advanced years. This interesting observation has often been debated and is briefly mentioned because it is relevant to the current theory.

**Figure 4:**
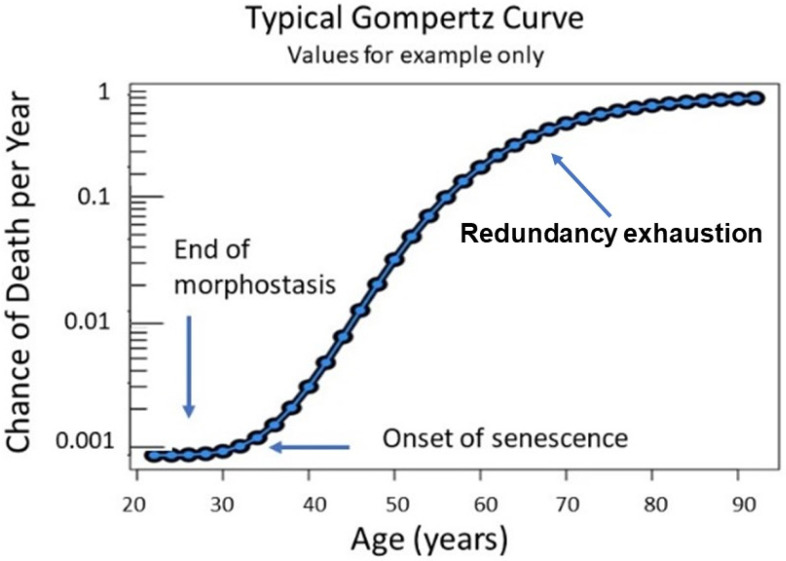
The Gompertz “death curve”. A representative plot of the Gompertz function showing the initial, rapid ascent upon initiation of senescence following morphostasis. Slope increases sharply as age-related death increases exponentially until late in life, when its rate decelerates due to exhaustion of morphostasis regulatory redundancy. Reproduced with modification from *Willis Eschenbach (2020) The Math of Epidemics, Figure 3*. https://wattsupwiththat.com/2020/03/13/the-math-of-epidemics/ with permission under terms of the Creative Commons Attribution 4.0 International License (http://creativecommons.org/licenses/by/4.0/).

Plots of Gompertz function begin in humans at about 30 years of age and are sigmoidal. However, unlike a simple logistic function in which both asymptotes are approached symmetrically, the left-hand or lower value asymptote is approached more rapidly than the right-hand or future value asymptote which represents a deceleration of the aging rate due to exhaustion of morphostasis regulatory redundancy and unimpeded effects of any remaining maintenance process (Figure 4). 

This special case of Richards curve [94] provides a clue to the nature of existing conditions before senescence begins and during the late stages of aging. Although the complete Gompertz function represents a correlation of aging with death, its abrupt initiation suggests that senescence is “released” from suppression as 30 years of age approaches. In contrast, since loss of redundancy has been suggested as a possible driving force of aging [67], its slowing toward the end of life could be due to redundancy exhaustion and any continuing effects of maintenance.

The Gompertz equation adequately represents progression of age-related adult mortality, but not that of younger individuals [95] when death due to aging is uncommon. Makeham [96] complemented the Gompertz function by including an age-independent term. The combined functions, commonly referred to as the Gompertz–Makeham (G-M) law of mortality, describe the frequency of deaths as a function of age in human populations throughout life beginning with a span of ages from birth to 20 years (presumed duration of the DP) and ending with age-related deaths from 30 until approximately 90 years and beyond. This pattern of age-related mortality provides considerable information about intrinsic factors underlying the emergence of senescence and the subsequent acceleration of aging [34]. 

Reliability models based upon defective redundant systems could explain why mortality rates of biological organisms increase exponentially with age as described by the Gompertz equation [67]. While differing in process from that described by Gavrilov and Gavrilova [67], the aging mechanism proposed in this theory is compliant with their suggestion that, as components of a redundant regulatory system such as that needed to sustain morphostasis are lost, the failure of the whole system (the organism) occurs at an exponential rate (during morpholysis). 

**Figure 5:**
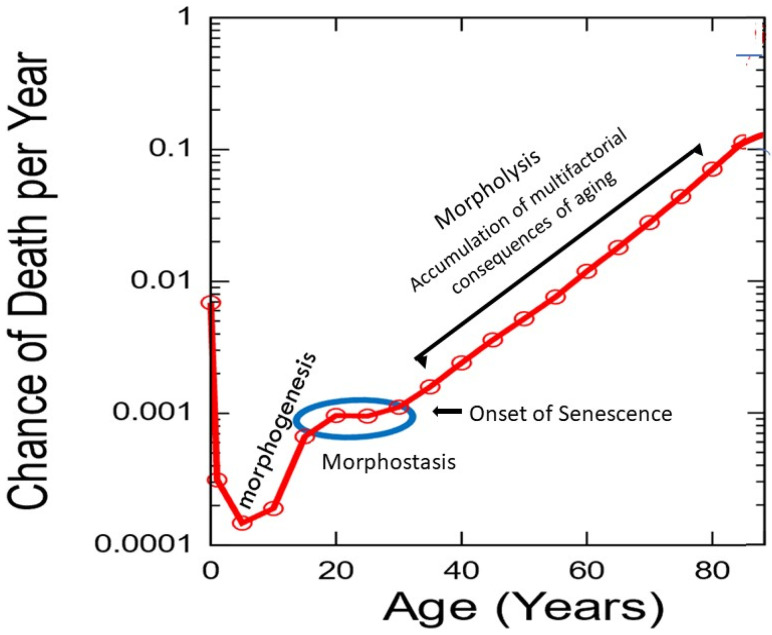
Plot of the Gompertz–Makeham “law of mortality”. Post-maturational “morphostasis” (circled) lasts in humans for approximately a decade between somatic maturation (completion of morphogenesis) and onset of exponentially increasing risk of death (morpholysis). When graphed, the G-M law displays a nearly horizontal line, representing the period of human morphostasis beginning at age 20 years and approaching the onset of age-related mortality at age 30. This figure in Volume 54, Number 14 *United States Life Tables*, 2003 by Elizabeth Arias, Ph.D., Division of Vital Statistics, is in the public domain and is allowed to be reproduced and modified without permission.

While the Gompertz segment of the G-M plot receives most attention, the age interval from 20 to 30 years is essentially ignored (Figure 5). 

This unique post-developmental segment of life represents a time when maturity and maximal stability are achieved during young adulthood, when most physiological processes are at peak performance capability. It is also contiguous with the end of development and the beginning of senescence when natural selection is operable but rapidly loses strength. The absence, or extremely low frequency of, age-related death during this decade has significant evolutionary benefit regarding reproduction, for which its underlying mechanism was selected as previously described. Thus, morphostasis is distinctly different from somatic maintenance.

## 15. Shroot’s Metaphor-Linking Development to Aging

The dynamic processes of development and aging have been traditionally considered as occurring sequentially, transitioning from the former to the latter at maturity. In humans, thirty years of age has been designated as that transition point based upon the completion of optimal structural development (maturation of male and female somatic characteristics) and the acquisition of peak physical/athletic performance capabilities [23]. 

The sequential phases of development and aging have been described metaphorically as a “hill”, the confluence of which is the apex. The metaphor assumes ascending and descending segments to represent the incremental growth of development and the decremental decline of aging, respectively [97].

Development is presumed to end at the apex after which senescence immediately begins. Thereafter, the soma undergoes a monotonic decline and loss of reserve that rapidly proceeds at a rate of about 0.5 and 1.0% per year, depending on the individual and the organ system being studied. The descent eventually leads to death from “old age” when physiological losses cross the minimum threshold for system autonomy [98]. The supposition that development and aging occur sequentially raised the “*long-standing, but increasingly relevant question; how are the two related?*” [99,100,101]. 

If development and aging were to occur sequentially, as described metaphorically by Schroots [97], the Gompertz function should begin at 20 years of age, immediately upon completion of the DP when somatic construction presumably ends. However, the mortality slope of the developmental curve abruptly stops rising in humans at about that age, remaining nearly horizontal thereafter (Figure 5). Thus, Shroot’s metaphor does not recognize that the G-M plot contains an intermediate stage when the soma exists in a state *neither of development nor aging* (Figure 5). If it did, the obvious answer to “how are development and aging linked?” would be through morphostasis, following significant change in the regulatory dynamics that previously guided morphogenesis. This interval of peak vitality in the absence of aging that bridges the life stages of development and senescence lasts approximately half as long as the time from conception to completion of somatic construction. The assumption that aging is ambiguous during this period of life can be tested by using the epigentic clock; however, this has not yet been attempted (Steve Horvath, personal communication, 13 July 2019; “*we did not yet look at it carefully because we don’t have good data from people younger than 30*”.)

Aging is delayed during morphostasis, but only temporarily, because its mechanism for stochastic damage repair is error-prone, causing failure within a few years due to accumulation of genetic damage, epigenetic alteration, and progressive loss of morphostasis regulatory redundancy.

Morphostasis ends with the gradual onset of senescence resulting from stochastic damage that accumulates to critical mass toward the end of the “plateau period”. This changes its initial conditions and, due to determinative chaos, its outcome, which ends the stage of non-aging somatic stability.

The third phase begins at approximately 30 years of age, with the onset of senescence and exponential progression of aging. As the end or morphostasis approaches, the beneficial effects of purifying negative selection is declining, thereby allowing DNA damage and subsequent epigenetic influence to alter initial conditions of the redundant morphostasis regulatory mechanism. Thus, regulatory behavior changes from causal determinism to deterministic chaos, which initiates senescence and subsequently accelerates morpholysis.

## 16. Senescence and Accelerating Rate of Aging

The present theory describes an evidence-based, evolved mechanism that initiates senescence and drives the exponential progression of aging in humans and other mammals. The reason for excluding other taxonomic groups is not to preclude the possibility of the mechanism applicable to them, but rather because it assumes an evolutionary requirement for continuation and redundancy of the last morphogenetic stage into young adulthood. It was selected to temporarily prevent aging and ensure parental vitality or provide opportunity for intergenerational transfer [102] in species whose offspring require nurturing, which does not occur universally, for example in some reptiles.

Prior to describing the specific aging mechanism, popular concepts in the multifactorial process of aging were reviewed. In most cases, the concepts were drawn from studies of older adult, organisms, which is the primary reason that the cause of aging has remained elusive.

During the 1990s, the discovery of a daf-2 gene mutation that doubled the lifespan of *Caenorhabditis elegans* led Kenyon to suggest the existence of “*a universal regulatory mechanism*” that determines the duration of life [103,104]. This was a seemingly logical assumption since daf-2 regulates a variety of physiological process at various stages of life by exerting control over many other genes. However, despite altering the gene’s expression and changing rates of aging, similar changes were reported by many investigators who altered expression of other genes and processes, leading to the conclusion that *aging is multifactorial*. As a result of the apparent complexity of the aging process, the concept of a unique “*regulatory mechanism*” was shelved as it seemingly became more obvious that singular control of such a multiplex process did not exist. Kenyon proposed that quantifying, analyzing, and understanding the aging process would require multi-layer and multi-tissue analysis, making it exceedingly difficult to accomplish that objective [105].

In agreement, Liochev claimed that the mystery of human mortality will never be solved because “*aging results from a significant number of causes*”, such that “counteracting one or several of them would make little difference” [106]. Data showing the widespread accumulation of molecular and cellular damages that interact in complex ways at various somatic levels suggested that many aging theories are simultaneously true [107]. Thus, testing them is the current pursuit of most aging research based upon the assumption that such efforts could lead to interventions that slow or alter aging. Maynard et al. proposed that through efforts to understand how DNA damage leads to aging, therapeutic approaches to prevent it are central to extend healthy life [108]. This shift in focus from basic research to therapeutic application is now dominant because the presumed multifactorial nature of aging is thought to increase susceptibility to a wide range of pathologies.

This widespread opinion directed research efforts away from the basic question of how aging occurs to manipulating its rates for therapeutic application in geriatric medicine [109]. Hayflick [110] opined that failure to distinguish biogerontology from geriatric medicine is the most serious impediment to understanding the aging process [26]. He stated “*There is a continuing belief that the resolution of age-associated diseases will advance our understanding of the fundamental aging process. It will not*” [110]. “*Just as the resolution of childhood pathologies…did not advance our understanding of childhood development, neither will study of the aging phenotype reveal the mechanism of aging*” [109]. This author agrees and describes a single mechanism that occurs before aging begins. It suggests that “why and how” senescence starts during the latter half of the post-morphogenetic decade, which exponentially increases aging thereafter. Prior to further discussion of that mechanism, a brief review of current findings and opinions derived from study of the aging phenotype is offered.

## 17. Misleading Premise of Current Theories

Most importantly, if not all damage theories consider that some of the maladaptive conditions associated with the aging phenotype are causal of it, they assume senescence *a priori*. If, as suggested, aging results from synergistic expression of ongoing, multifactorial, and maladaptive events that accelerate its progression, then they must have been in existence before the cooperative effects actually occur. In other words, the premise that synergy is a “*most significant cause of aging*” [106,111] is flawed because, like many other aging theories, it requires senescence to have already happened. It does not explain how the aging process begins in the first place.

A common error in logic shared by most biogerontologists relating to whether they favor programmed or non-programmed theories is that *their focus is upon the aging phenotype itself*. This may seem reasonable, but it is not. Generally, when seeking the cause of an effect, one would not expect to find it after the fact. It is more logical that an action will precede an outcome. This is the case for the cause of aging. Regarding programmed aging, the cause is obviously a program, which must be designed in such a way so as to produce the desired outcome, i.e., ultimately death. While the process (a program) is appropriate, its objective (to end organismal life) violates evolutionary mechanics and, therefore, is untenable. Alternatively, damage-based theories tend to lure investigators to the outcome within which they search for a cause. Aging is a somatic catastrophe that is expected to contain all sorts of damages, malfunctions, disorders, and diseases. However, diseases or pathophysiological disorders are not often considered as basic causes of aging because they are recognized as being products of pathogens or physical degeneration. In other words, they are consequences not causes. Unlike disease, molecular or metabolic stochastic damage is more enticing because it often occurs, at least initially, without overt pathological symptoms and, thus, as seemingly causal candidates for aging itself. Indeed, DNA damages and epigenetic abnormalities are found within the aging phenotype; however, if this is the case, the question concerns whether any can initiate aging in isolation from it. The answer is yes, consistent with Schumacher et al.’s statement that “*it remains unknown whether ageing has a unifying causal mechanism or is grounded in multiple sources.*” [112]. A central concept of the current theory is that the study of organisms that are already experiencing aging is somewhat illogical since senescence has already occurred and the dynamic process of morpholysis is proceeding.

As a metaphor for the misunderstanding of aging’s cause and effect, imagine a large house standing alone in an open field, unprotected from environmental assaults and out of view by rational beings. Suddenly, the building is severely buffeted by a tornado force wind that appears from nowhere, and then immediately disappears. The building’s structural integrity is totally compromised by the assault to the extent that it is rendered unstable and begins to collapse. Thereupon, observers suddenly appear on the scene to witness the building’s progressive disintegration, unaware of the environmental trauma it previously experienced. They wonder, “*what’s causing this structural collapse”? Is it the broken rafters and trusses, the cracked foundation and split supporting beams, opening of a subterranean sinkhole, isolated shifting of a microtectonic plate below the basement, aggressive attack by wood boring insects…anon, anon*.”. This ridiculous metaphor is intended to explain how, in biology, the aging body, like the collapsing building, presents its current condition to an observer, as opposed to the prior experience responsible for causing it. Like the collapsing building, a multitude of seemingly possible causes can be observed in the body as it ages; however, none are sufficient in isolation to be the primary reason for its all-consuming and progressive disintegration. Nonetheless, there is the seemingly universal tendency to look at events that occur during aging as a cause of it, as opposed to a consequence. As a result, there is a huge store of information on DNA damage, epigenetic modification, metabolic and mutational events, etc., which all occur during aging. Despite examination and manipulation of the “impressive diversity”of its correlates, only the rate of aging can be altered. Somatic deconstruction is never prevented, and youth is never sustained. This barrier exists for two reasons. Firstly, none of the hallmarks of aging are its primary cause. The second preferable reason is that current technology to selectively block expression of the actual regulatory events that inadvertently initiate senescence and accelerate aging thereafter do not exist. So, for the present, describing the existence of a functional mechanism that causes aging may be sufficient incentive for such technology to be developed.

Consistent with this objective, Ryosuke et al. [44] argue that despite an amalgam of multiple, random detrimental effects being generally accepted, the identification of mutations and other genetic structural changes that affect longevity suggest that particular cellular pathways affect aging. They then recognized that the challenge of understanding the aging process is to determine the underlying mechanistic bases that regulates it. As will be described below, DNA double-stranded break (DSB) damage and the resulting regulatory gene structural change and subsequent transcriptional dysregulation within the developmental regulatory process directing morphostasis are directly involved in the underlying mechanism of aging. 

With humility, the author’s previous comments were not intended to besmirch the opinions of those who search within the aging phenotype for its cause or imply that their findings are not of value. Those efforts have generated extensive information on molecular damage and maladaptive metabolic and physiological changes that occur during aging. However, searching for the basic underlying mechanism of aging within its phenotype is folly that will ultimately produce frustration and/or abandonment in favor of efforts to exploit more pragmatic and therapeutic approaches to aging research. In contrast, identifying the mechanism by which aging emerges and progresses may create a means for managing the plethora of pathologies associated with it, before they develop into frank disease states. In other words, the shift in research from applications intended to treat age-related disease to the prevention of it through comprehension of the primary cause may result from understanding the basic mechanism of aging. Accordingly, a central concept of the current theory is that when the effects of DNA DSB damage the morphostasis regulatory mechanism and escape the constraints of NS, maladaptive changes occur in the structure of regulatory ncDNA. This effect alters epigenetic influence over determination of the regulatory mechanism’s “initial conditions”, thereby changing its dynamic behavior that disrupts appropriate regulatory oversight to ultimately initiate senescence.

Of note is that the effects of both DNA damage and epigenetic influences that alter expression of initiating regulatory genes and have primary roles in aging also occur stochastically within aging organisms due to generalized breakdown of local regulatory oversight and control. However, identifying primary participants in the mechanism for aging, as described within this theory, requires an exertion of their maladaptive influence before senescence emerges in the young organism. Prior to describing that proximate mechanism of aging, selected data on aspects epigenetic and DNA damage derived from study of the aging phenotype, and thereby not representing its causal mechanism, are briefly compared and contrasted. 

## 18. Epigenetics

Epigenetic dysregulation is considered a key hallmark of the aging process [113]. However, it is not exclusively responsible since genetic impairment and non-genetic, i.e., epigenetic factors, jointly contribute to longevity [114]. If so, this possibility raises a “chicken or egg” question of whether changes in the activity of epigenetic enzymes influence the expression of critical longevity genes or whether alterations in the longevity genes drive large scale epigenetic changes in the genome [113]. Based upon the observation that single-point mutations in epigenetic enzymes dramatically alter the lifespan of lower organisms, it is possible that epigenetic modifications drive age changes. However, since there are many redundant enzyme systems in more complex higher organisms, Sen et al. [113] concluded that having a few genes as drivers of age-related changes is too simplistic an idea. Instead, large-scale changes due to environmental stimuli or nutrient availability were suggested to be primary factors. It is noteworthy to reiterate a point previously made, i.e., that this conclusion was drawn from study of aging subjects. 

In any event, cellular phenotypic diversity is explained by epigenetic influences that modify genetic expression. Optimal gene expression is maintained through rounds of cell division due to key, stable epigenetic patterns of DNA methylation and chromatin-based regulation. However, the role of DNA methylation is complex, as it affects gene expression depending upon CpG context [115].

Evidence that CpG island methylation is a true epigenetic mark derives from its stability and self-perpetuation through cell divisions. For example, promoter, gene body, and intergenic methylation are associated with gene silencing, variable effects on gene expression, and enhancer regulation, respectively [116]. However, in other contexts, such as that involving the developmental regulatory mechanism, epigenetic profiles are more dynamic, sometimes *following changes in gene expression rather than causing them*, thereby not reflecting the stability of a true epigenetic state. This variation is of primary importance in the mechanism subserving the emergence of senescence from morphostasis, since structural alteration of the initiating genetic component that alters subsequent outcome and, thus, the epigenetic state is proposed to specifically result from DSBs. Central to the theory is the importance of mechanistically distinguishing between stable epigenetic expression needed to maintain somatic homeostasis from its dynamic expression in the developmental regulatory mechanism during the various stages of morphogenesis, morphostasis, and morpholysis.

We now consider epigenetic changes in the aging phenotype. Methylation changes occur progressively and linearly, such that hyper- and hypo-methylation within the soma are consistent with a gradual change away from baseline or “drift”. This behavior differs from that which is abrupt or programmed as during expression of the holistic regulatory mechanism. Aging “drift” is most likely due to stochastic errors which cause the imperfect maintenance of epigenetic marks. Based upon these, it is possible to estimate organismal age, by measuring DNA methylation “drift” in peripheral blood and other tissues [117,118]. In this context, a methylation change simply defines differences between two somatic states as in comparing young individuals with old ones. It does not necessarily imply active participation in the regulatory mechanism, nor functional consequences or primary pathology. However, shifts in global methylation can be indicative of physiological and/or pathological changes [119,120]. Since age-associated phenotypes can be ameliorated by epigenetic remodeling during cellular reprogramming, epigenetic dysregulation qualifies as an active participant in driving mammalian aging, without necessarily initiating it [120].

During the early stages of morphogenesis, when a stable environment that minimizes molecular and cellular damage exists, cells undergo spatiotemporally orchestrated differentiation that ultimately generates all the cell types of an adult organism. However, as the organism ages beyond adulthood, the continuous and progressive loss of morphostasis redundancy exacerbates aging. As a result, efficacy of the mechanisms responsible for minimizing cellular damage declines during aging, eventually resulting in an organism’s inability to maintain homeostasis.

Indicative of this process are the many epigenetic marks, such as DNA methylation, post-translationally modified histones, and chromatin, which all change during reprogramming and thereby become dysregulated during aging. By not considering that a primary mechanism of aging also involves genetic and epigenetic elements that regulate morpholysis, epigenetic dysregulation during aging, i.e., not preceding it, has emerged as a hallmark of the process [113]. The important difference between these two processes is that aging is driven by a deterioration of the primary mechanism; thus, global epigenetic dysregulation is a consequence of the aging process, not a cause. 

Many of these age-related somatic changes can be ameliorated by increasing the levels of histones, strongly implicating direct transcriptional consequences of histone loss [121]. Genomic stability and chromatin structure are closely intertwined. The latter not only regulates accessibility of DNA damaging agents to the genome, but also participates in critical signaling roles for DNA lesions and their repair [122]. Since chromatin plays a critical role in regulating genomic stability and gene expression, it is possible that such changes may be caused by the global alteration of chromatin structure during aging, as evidenced by the loss of heterochromatin in human cells [123] to a wide-spread reduction in histone levels during mitosis. 

To summarize this brief review, epigenetic changes that are directly related to expression of the regulatory mechanism for oversight of various life stages, e.g., morphogenesis, morphostasis, and morpholysis, are different from those associated with somatic maintenance and function, especially during aging.

## 19. DNA Damage

DNA damage and mutations have long been considered key causal events and potentially universal participants in the aging process. Accordingly, putative genome maintenance systems exist to sustain longevity by repairing damage or removing cells, whereby DNA damage is beyond repair [124,125].

Data from humans and animals experiencing some degree of aging support the view that there are many causes, including mitochondrial dysfunction, free radical damage, telomere shortening, cellular senescence, stem cell depletion, and destabilization of energy homeostasis [108].

However, it is generally agreed that none of these effects independently represent the cause of the aging, but rather that they interact in complex ways to erode genomic maintenance by accumulating DNA damage which has adverse phenotypic consequences in adult organisms [126,127]. Supporting that proposal is evidence that genome rearrangements and structural variations, including mutations, deletions, and translocations, occur in somatic tissues and increase with age [128,129]. 

Because it is a major target of age-related cellular harm, and since human premature aging syndromes are caused by progressive genome instability [45], DNA damage has long been considered a hallmark of aging and a potentially universal participant in its causal mechanism [124,125]. 

DNA damage results from various intrinsic and extrinsic insults to the soma, generating in humans as many as fifty thousand lesions per day per cell [130]. These lesions include base modifications, single-strand breaks (SSBs), double strand breaks (DSBs), and interstrand cross-links (ICLs). Since DNA damage can interfere with transcription and replication, it can erode cell function and lead to death [127]. 

The negative effects of DNA damage on genome destabilization are exacerbated by its accumulation due to the functional decline of the DNA damage repair mechanism as aging progresses [131]. In animal models, defects in DNA repair are associated with significantly shorter life spans than long-lived mutants that display increased repair capacity. Similarly, some heritable diseases linked to defective DNA repair or damage processing characteristically display premature aging and early death. Comparable DNA repair defects produced in murine models by genetic modification caused age-related disease phenotypes to emerge [132]. The opinion that accumulated DNA damage associated with defective repair assaults genome integrity is supported by the existence of ubiquitous, dedicated, and energetically expensive repair mechanisms. These exist to correct DNA damage that can disrupt cellular homeostasis and presumably cause aging. Thus, reference is made to recent, relevant reviews for a more comprehensive background on the relationship between DNA damage and aging [112,133].

Although erosion of genome integrity by DNA damage is favored as a basic cause of aging, there is little consensus on which kind is primarily responsible in raising the question “*does any one type of damage play a predominant role in causing aging, and if so, which?*” [108]. White and Vijg proposed that DSBs are responsible [134]. 

## 20. Double-Stranded Breaks

DNA DSBs display several characteristics that make them causally relevant to aging [124]. They occur at a rate of about 10 to 50 per cell per day depending on cell cycle and tissue. However, their frequency increases with aging, and they are highly toxic, making them capable of having severely adverse consequences [135]. Defects in their repair underscore the relevance of DSBs to aging by linking such repair with age-related chromatin changes. If unrepaired, they can cause cell death or, *if incorrectly repaired, they may produce gene rearrangements* [136]. It is this effect of DSBs on gene structure that is directly applicable to disruption of morphostasis redundancy, which initiates senescence in humans as they approach 30 years of age.

DSBs can result from cellular exposure to DNA damaging agents; however, *they also occur in a programmed manner during development*, generated to initiate recombination between homologous chromosomes during meiosis [137]. They also occur as intermediates during developmentally regulated rearrangements, such as V(D)J recombination and immunoglobulin class switch recombination. When occurring during youth and under normal physiological conditions, adverse outcomes of DSB processing are rare. In fact, they can occasionally be beneficial when involving apoptosis and cellular senescence, which can efficiently remove irreversibly damaged cells or constructively participate in the developmental process, respectively [138,139].

The cumulative maladaptive effects of DSBs, which are different from those specific to initiating the aging mechanism, emerge during aging itself, causing atrophy, inflammation, and immunosenescence. Undoubtedly, these adverse conditions resulting from DSB accumulation are consequences of aging. More relevant to the theory is that DSBs are intrinsic to various biological processes. For example, transcription is a key substrate for translocations, deletions, and amplifications associated with aging and various cancers [140,141]. 

Changes in DSB processing and repair can *independently* cause aging, as evidenced by the effects of non-specific, radiation-induced lesions in rodents and humans, as well as by treatment with high doses of anti-cancer agents or by their enzymatic induction [50,134]. These observations suggest that, unlike other DNA damage, DSBs may be capable of driving multiple age-related phenotypes, as part of the aging mechanism.

DSBs also promote epigenetic drift by triggering signals that recruit epigenetic regulators away from gene promoters to the DNA break site [142] and are consistently linked with accelerated aging if defectively repaired [143,144].

In contrast, a survey of long-lived species showed that the efficient repair of DSBs or the inhibited rate of damage was highly correlated with longer lifespan [145]. Thus, DSBs threaten cell viability by compromising integrity of the genome as well as the epigenome.

## 21. DNA Damage Response

Following generation of a DSB, the cell initiates a DNA damage response, consisting of highly orchestrated series of events, during which the two broken DNA ends are reconnected [146]. The two predominant mechanisms for DSB repair are homologous recombination (HR) or non-homologous end joining (NHEJ). In mammals, the DNA damage response (DDR) commonly involves the NHEJ pathway which does not require homologous ends to be joined after minimal processing by nucleases [147]. However, DDR using NHEJ, which is the primary repair mechanism in mammals [148], is error-prone, sometimes causing structural variations, including small base pair deletions and translocations [149].

The improper DSB repair exacerbated with advancing age is exacerbated by the increasing numbers of DSB foci that occur in multiple tissue types, reflecting a possible increase in the accumulation of unrepaired DSBs due to progressively delayed repair events. Delay may occur as a consequence of an inherently limited capacity to process DSBs. The declining age-related capacity of NHEJ throughout the body has been demonstrated in knock-in reporter mice [150]. These changes in DSB processing and repair can *independently* cause aging, as evidenced by the effects of radiation-induced lesions in rodents and humans, as well as by treatment with high doses of anti-cancer agents. More definitively, mouse models to evaluate the effects of DSBs demonstrated that patterns of liver-related aging pathology were increased, similar to those seen in naturally aged mice [134]. More recently, a transgenic mouse model using the I-PpoI restriction enzyme to induce DSBs [50,151] caused premature holistic aging, discussed as supporting data for the proposed aging theory involving damage to the morphostasis regulatory process. These observations suggest that, unlike other DNA damage, DSBs are independently capable of driving multiple age-related phenotypes as part of the aging mechanism. Furthermore, the quality of DSB repair responses tends to degrade with age, causing increased genome structural variation in older mammals. The diminishing quality of DSB repair that normally occurs, as well as following long-term exposure to agents causing their induction, is associated with premature aging.

## 22. DSB Misrepair—Effects on Regulatory Gene Structure

The improper repair of DSBs by error-prone NHEJ can cause structural variations [148]. Genome rearrangements and structural variations, including mutations, deletions, and translocations, occur in somatic tissues and increase with age [128,129].

The relevance of DSB-induced mutagenesis to the theory is its potential to change the structure of coding genes, causing transcriptional dysregulation, similar to Huntington’s disease (HD) [152]. The same effect can occur in ncDNA, making the maladaptive effect of DSBs on transcriptional dysregulation relevant to **the** changing behavior of the regulatory mechanism directing morphogeneis as humans approach 30 years of age [153].

Consistent with this concept is the decrease in transcriptional coordination in aging cells associated with high stochastic mutational load and radiation-induced DNA damage that produces DSBs. Thus, transcriptional dysregulation resulting from stochastic events may represent a central and causal attribute of dysfunction in the morphostasis regulatory mechanism, thus leading to aging [154].

**Figure 6:**
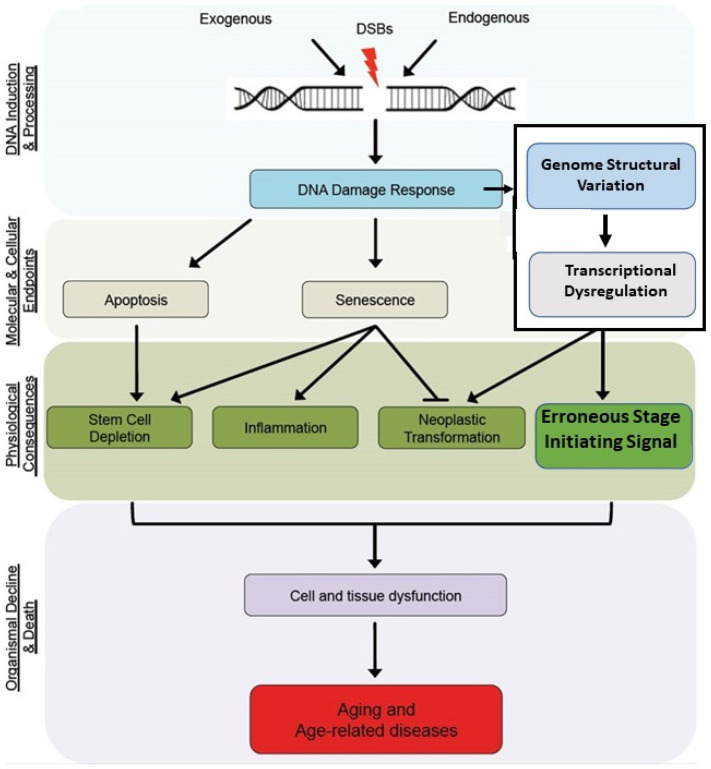
Effects of DNA double-strand breaks on structural change in genetic components of the morphostasis regulatory mechanism. Effects of DNA DSBs on cells include apoptosis, senescence, and structural change. The latter is specifically relevant to the theory because genome structural change is proposed to be the pivotal effect of DSB damage that disrupts morphostasis regulatory redundancy. Reproduced and modified with permission from [134].

A clue to the link between DSB repair and changes in the regulatory mechanism that initiates senescence was provided by White and Viig [134] who summarized data, demonstrating the effects of age on the repair of DSBs. Based upon the observations that genetic defects in DSB repair are associated with premature aging in humans and mice, and that aging accelerates following exposure to radiation, chemotherapeutic agents, or genetic constructs with restriction enzymes, DSBs may play a causal role in driving aging. The authors speculated that DSBs do so through other molecular and cellular end-points, as well as genome structural variations [Figure 6].

These can cause detrimental physiological consequences over time, leading to the progression of aging, disease, and death. The description of the possible products of DSB misrepair has been modified to specifically focus upon structural change in ncDNA that ultimately alters initial conditions for morphostasis regulatory control, thereby changing its behavior to chaotic from determinate, and eroding redundancy with each progressive regulatory cycle in which DSB damage and misrepair occurs.

Because normal DSB repair responses tend to decline during aging, genome structural variations increase. Likewise, multiple premature aging phenotypes can occur very early in life when DSBs are experimentally induced, providing evidence of a cause-and-effect relationship [134]. Specifically, the improper repair of DSBs resulting in maladaptive structural changes in ncDNA could introduce transcriptional dysregulation of regulatory ncRNA to subsequently change initial conditions of the morphostasis regulatory mechanism. This effect shifts regulatory behavior from deterministic to chaotic which then erodes strict redundancy of morphostasis, thus initiating senescence and accelerating the rate of aging. 

## 23. DSB Involvement in the Theoretical Aging Mechanism

As shown in Figure 6, the misrepair of DSBs by the DDR can result in genome structural change. An assumption of the theory is that structural changes in genes, setting initial behavioral conditions of the regulatory system during latter morphostasis when the strength of NS wanes, will change the outcome of the segment within which it occurs. Change in outcome will modify epigenetic influence on those affected gene’s expression during the next cycle. Since such change alters initial conditions of subsequent cycles, the determinative behavior of the regulatory mechanism will eventually become chaotic. The change in regulatory behavior will instigate a loss of morphostasis redundancy and thereby initiate senescence. The progressive accumulation of genetic structural change in other parts of the global morphostasis regulatory mechanism exacerbates chaotic behavior, increasing the loss of regulatory redundancy. Then, the driving force of DP regulation exponentially accelerates aging since the number of erroneous regulatory commands approach and exceed those that are correct. Besides explaining why the rate of aging rapidly increases, this effect also explains why the progression of aging appear to be programmatic.

Supporting evidence for this proposal derives from experimental data published by Hayano et al. [50], summarized below.

## 24. Supporting Evidence

Hayano et al. [50] created a transgenic mouse system called Inducible Changes to the Epigenome (ICE) using mature mice ranging in age from 3 to 6 months. The mice selected for study were at “*a life phase equivalent for humans ranging* *from 20–30 years*” [155]. Thus, the study was conducted on mice whose physiological ages were comparable to those of humans during the decade of morphostasis.

The study was designed to determine how experimentally induced DSBs, in conjunction with correlative changes in the epigenome, affect organismal aging. Non-mutagenic DSBs were created in non-coding regions of DNA at frequencies only a few-fold above normal background levels using a homing endonuclease (l-PpoI), encoded by a selfish genetic element from Physarum polycephalum. Initially, ICE mice did not display physiological or molecular changes, nor did they display any advancement of the epigenetic clock [156]. No discernable differences in behavior, activity, or food intake were observed during the three-week l-Ppol induction period, whereas visible differences were observed after one month. When compared with controls, ICE mice displayed common features of middle-aged, wild-type mice, including slight alopecia and loss of pigment on their feet, tails, ears, and noses. Four to six months later, the ICE mice displayed significantly accelerated physiological aging of skin, eye, muscle, and brain. Transcriptional changes associated with acceleration of the epigenetic clock estimated the rate of aging to be about 50% faster than controls. By 10 months of age, gene expression patterns and DNA methylation in ICE and control mice showed that dysregulation of skeletal muscle genes from significantly younger treated mice was positively correlated with that of twenty-four-month-old wild types. Resulting molecular changes in the epigenome included histone modifications, DNA compartmentalization, smoothing of the epigenetic landscape, and loss of cellular identity.

Thus, the cutting of ncDNA in young adult mice rapidly changed their epigenome, which was associated with the initiation and acceleration of aging. Thus, the authors proposed that their findings did not support the generally held damage-based theories that aging is a random multifactorial process; however, instead, it was argued that it is non-random and “*potentially driven by reproducible and predictable epigenetic changes*”. Based upon the relocalization of chromatin modifiers hypothesis [142], the authors suggested that the repeated misrepair of DSB damage progressively altered the epigenetic landscape to the point where cells remained in a chronically stressed state, eventually disrupting their cellular identity.

While admitting to not knowing the specific reason that aging was accelerated in their model, Hayano et al. speculated that a general mechanism, involving DNA damage, chromatin, and/or transcriptional networks, triggered a feed-forward cascade of “deleterious events” [50], which resembled accelerated aging. Consistent with the concept that aging is never due to changes in a single local system, but instead to generalized deterioration of the soma, they proposed that “*DNA damage/repair based loss of epigenetic information* [throughout the body] *is an upstream cause of mammalian aging*”. The ICE data support that premise; however, identification of a naturally occurring process(s) by which the senescence-causing scenario plays out is lacking from their conclusion. This is presented in this paper’s theory. Nonetheless, they provided a preliminary explanation for how a molecular mechanism could cause holistic aging in mammals, suggesting that DSB repair alone accelerates aging at physiological, histological, and molecular levels, as well as accelerating the epigenetic clock.

The authors stated that accelerated aging in the ICE mice was not due to mutations since the number of potentially active genomic elements for the regulatory mechanism was enzymatically eliminated. This view is inconsistent with the report of White and Vijg [134] and the assumption that structural change is one possible effect of DSB damage repair that disrupts normal progression of the morphostasis regulatory mechanism. However, enzymatic cutting of genomic constituents of the regulatory mechanism is functionally the same since the process effectively reduced the number of valid components of the regulatory mechanism that would occur either by mutation or by physical elimination. Nonetheless, there is an operational difference between the progression of morphostasis regulatory expression in the experimentally induced and normally aging mice. The difference involves negative (puifying) selection which participates in the latter condition but not in the former. As previously described, natural aging involves structural change in genetic components of the morphostasis regulatory mechanism. If these were not removed, they would immediately change the initial conditions of the next morphostasis cycle, thereby interrupting system redundancy to some extent. This concern is not immediately relevant to the ICE mice, since their treatment did not cause mutations but instead reduced the number of genetic components participating in redundant morhostasis regulation, according to Hayano et al. [50]. Thus, purifying selection is needed to sustain morphostasis during the natural life span. However, it paradoxically also contributes to aging by reducing damaged components of morphostasis, inadvertently causing eventual loss of redundancy.

Hayano et al. [50] were also to answer some important questions that are addressed in the current theory. For example, they could not explain how “*seemingly random events involved in their experimental process appear to behave as if they were part of a program*” since instantaneous global damage to ncDNA and its associated negative impact on the epigenome caused aging in a seemingly programmatic and naturally occurring progression, albeit at faster rates. However, the authors did not argue that their findings supported the concept of “programmed aging”, even though they recognized that the effects of treatment on progression of the aging phenotype appeared to be programmatically expressed. Instead, they proposed that instantaneous, widespread, and increased, but non-mutagenic, levels of DNA damage *accelerated* the epigenetic clock, consistent with increased rates of physiological, cognitive, and molecular aging in their experimental animals. The study identified a molecular driver of epigenetic change during aging and provided convincing preliminary evidence that it affects the aging process. In lieu of providing a detailed mechanistic answer, the authors proposed that DNA damage, independent of mutations, drives the “aging clock”, thus explaining why morpholysis proceeds through a predictable series of molecular and physiological changes. Furthermore, they proposed that their idea provided evidence of a holistic senescence mechanism, despite the random occurrence of DNA damage anywhere in the genome.

Their logic that DNA damage and associated epigenetic changes drive the epigenetic clock implies that the aging process is directed, rather than being its “time keeper”. While there are no published data demonstrating that the “clock” is a driver of aging, Horvath and Raj [157] proposed an epigenetic clock theory that views organismal aging as an unintended consequence of developmental and maintenance programs. These, in turn, provide molecular footprints which act as the basis for age estimation by DNA methylation (DNAm). In other words, rather than being a driving force of aging, DNAm age is a “*proximal readout*” of all the events innate or otherwise that represent the root causes of aging and ultimately erode cell and tissue function. It is this author’s opinion that the epigentic clock, while serving as a proximal readout of aging’s progression, also reflects the multiple well-recognized consequences of advancing age within the body. Therefore, damage-related acceleration of the epigenetic clock is not the proximate cause described within the current theory; however, instead, it measures the results of aging itself that are widely recognized as being multifactorial. Because epigenetic clocks provide molecular correlates of chronological age in humans and other vertebrates, they could prove useful for evaluating rates of aging, as well as for the efficacy of interventions focused upon rejuvenation and extending lifespan [158].

## 25. Discussion and Conclusions

This theory resolves the century-long debate over whether the cause of aging is an evolved program to ensure determinative lifespan due to accumulated damage within the adult soma. Neither of the historic approaches provide a mechanism which can describe how aging begins and how it exponentially accelerates as mammals approach and transcend middle age.

Relevant to the mechanism of aging that occurs within the proposed process of morphostasis is the fact that the soma is under threat of constant assault by DSB damage to DNA. Presumably, such damage invariably occurs within the morphostasis mechanism, where determinative behavior is only maintained by the strict redundancy of regulatory expression. Since structural alteration is an effect of DSB damage, its occurrence in the regulatory DNA would change the outcome of morphostasis cycles. Since the outcome represents the epigenetic modifier of genetic expression during the subsequent cycle of the regulatory mechanism, the initial conditions of that component of the mechanism, wherever in the body it resides, would be changed. As a result, the strict redundancy of that segment of the morphostasis mechanism would be lost, thereby causing a slight change in overall mechanistic behavior to determinative chaos which would initiate senescence. However, because the accompanying effects of early damage are few, the outcomes of the mechanism retain predictability for a while. Thus, the onset of aging, as represented by an initiation of senescence from morphostasis that occurs in humans at about 30 years of age, is not immediately apparent by obvious phenotypic changes. However, the continued purifying negative selection of polymorphisms, resulting from DSBs within somatic cells over time, increases erosion of morphostasis redundancy, which amplifies the error and exacerbates chaotic behavior within the regulatory mechanism. The accumulation of DSBs within the mechanism occurs randomly within the soma; however, aging is also influenced by inherited and environmental factors that modulate its rate and characteristics between individuals. The combined effects undoubtedly account for differences in aging trajectories between them.

Subtle changes in physical appearance become more apparent during the fourth decade of life and increasingly so during the fifth, since the progressive loss of redundancy accelerates the rate of aging and thus accounts for its typical phenotypic changes. During these decades and beyond, dysregulation of the mechanism promotes disorder within metabolic and physiologic homeostasis control systems, resulting in the emergence and unambiguous expression of the “hallmarks of aging”, which are consequential of the condition, not causal. As a result of these maladaptive effects upon the aging condition, resistance to intrinsic disease significantly decreases, accounting for the common occurrence of cancers, arthritis, type 2 diabetes, cardiovascular disease, and any number of metabolic disorders when a person reaches their late fifties. Thereafter, surviving individuals progressively become frail, osteoclastic, less resilient, and more vulnerable to communicable diseases. Furthermore, increasing physical damage, such as fractures and internal injuries, result from falls or other accidents due to declining proprioception, vision, and hearing. Eventually, late in life, the exhaustion of the morphostasis redundancy mechanism occurs, allowing any remaining somatic maintenance processes to slightly decelerate the rate of aging. Finally, death is inevitable when tolerance to stresses and physiological fluctuations is no longer possible, causing internal entropy to accumulate as energy throughput irreversibly diminishes.

## Figures and Tables

**Table 1:** A summary of aging theory characteristics showing significant deficiencies in Programmed and Non-Programmed (Stochastic Damage) categories regarding lack of mechanism or program, respectively, in currently favored theories. In contrast, the Unifying, holistic theory of aging resulting from changing DP-based, organismal regulatory dynamics during the lifespan, contains both characteristic and thereby answer’s questions about the origin of progression of aging that are unanswerable by other two theories.

Aging Theories Compared and Contrasted			
Type	Unified	Programmed	Stochastic
Aging mechanism-Origin and Type	Developmental Program	Novel Program	Random Program
Expression separate from Developmental Program	No	Yes	Yes
Aging mechanism selected	No	Yes	No
Objective-Initiate aging and terminate individual life	No	Yes	N/A
Adaptive outcome–Population benefit	Yes	Yes	Yes
Violates individual benefits	No	Yes	No
Empirical evidence supporting molecular mechanism	Yes	No	Yes
Explains programmatic appearance of age progression	Yes	No	No
Explains acceleration and deceleration of aging	Yes	No	No

## Data Availability

Not applicable.
